# Robotic-assisted total knee arthroplasty in clinical practice: protocol for a randomised controlled trial

**DOI:** 10.1186/s13018-023-04101-z

**Published:** 2023-08-25

**Authors:** Filippo Migliorini, Nicola Maffulli, Luise Schäfer, Jens Schneider, Andrea Maria Nobili, Daniel Kämmer, Milosch Michalak, Andreas Bell

**Affiliations:** 1 Department of Orthopaedic, Trauma, and Reconstructive Surgery, RWTH University Hospital, Pauwelsstraße 30, 52074 Aachen, Germany; 2 Department of Orthopaedic and Trauma Surgery, Eifelklinik St.Brigida, 52152 Simmerath, Germany; 3 Department of Orthopaedic and Trauma Surgery, Academic Hospital of Bolzano (SABES-ASDAA), 39100 Bolzano, Italy; 4 Department of Medicine, Surgery and Dentistry, University of Salerno, 84081 Baronissi, SA Italy; 5 School of Pharmacy and Bioengineering, Faculty of Medicine, Keele University, Stoke on Trent, ST4 7QB England; 6 Queen Mary University of London, Barts and the London School of Medicine and Dentistry, Centre for Sports and Exercise Medicine, Mile End Hospital, London, E1 4DG England

**Keywords:** Knee, Arthroplasty, Robotic, CORI

## Abstract

Between 2 and 20% of patients who undergo total knee arthroplasty (TKA) report restricted motion and anterior knee pain. Non-optimal alignment of the implant components is a common cause of such complaints. Robotic-assisted TKA has been advocated to improve the accuracy of component positioning to match patients’ anatomy and biomechanics. However, the advantages of robotic surgery over conventional freehand TKA are still unclear. The present study is a protocol for a single-blind clinical trial in which patients will be randomly allocated to undergo either robotic-assisted TKA or conventional freehand TKA. A restricted kinematic alignment with medial para-stellar approach shall be made in all patients. The present study follows the SPIRIT guidelines. The primary outcome of interest is to compare robotic TKA versus traditional freehand TKA in terms of patient-reported outcome measures (PROMs), length of hospitalisation, blood values, blood transfusion units, and range of motion. The second outcome of interest is to evaluate the accuracy of component positioning of robotic-assisted TKA compared to the conventional freehand TKA.

**Level of evidence** Level I, randomised controlled trial.

*Registration* German Registry of Clinical Trials (ID: DRKS00030614).

**Supplementary Information:**

The online version contains supplementary material available at 10.1186/s13018-023-04101-z.

## Introduction

Total knee arthroplasty (TKA) aims to restore knee pain and function, and improve the quality of life of patients [1–3] with end-stage osteoarthritis (OA) [4]. However, between 2 and 20% of patients who underwent TKA reported restricted motion and anterior knee pain [1, 5, 6]. These complications could arise from a non-optimal alignment of implant components [2, 7]. In this context, the introduction of robotic-assisted TKA is supposed to improve the accuracy of component positioning and to match more precisely patients’ anatomy and biomechanics [8]. Robotic TKA has been introduced to improve component alignment [9–11], impacting favourably on postoperative pain, hospitalisation, long-term implant survival [12, 13], and patient satisfaction [14]. However, the advantages of robotic surgery over conventional freehand TKA are still unclear, and high quality clinical investigations on large scale are necessary. Therefore, a single-blind randomised controlled trial will be conducted to evaluate TKA using the CORI robotic system (Smith & Nephew PLC, London, Great Britain). The primary outcome of interest is to compare robotic-assisted TKA versus the conventional freehand TKA in terms of patient-reported outcome measures (PROMs), length of hospitalisation, blood values, blood transfusion units, and range of motion. The second outcome of interest is to evaluate the accuracy of component positioning of robotic-assisted TKA compared to the conventional TKA.

## Methods

### Study protocol

The present study shall be conducted in accordance with the SPIRIT 2013 statement (defining standard protocol items for clinical trials) [15]. All patients who will receive a TKA at the Department of Orthopaedic Surgery of the Eifelklinik St. Brigida in Simmerath (Germany) will be prospectively invited to participate in the present investigation. The recruitment will start on January 1, 2023, and will stop on January 1, 2033. The present study shall be conducted according to the principles of the Declaration of Helsinki. The shall authors receive no financial support for the research, authorship, and/or publication of this article. The protocol of the study has been prospectively registered and approved by the German Registry of Clinical Trials (ID DRKS00030614). Ethics approval has been received from the North Rhine Medical Council, Dusseldorf, Germany (ID 2022374).

### Participants

Patients who will agree to participate in the present study will be informed preoperatively of the purpose of the study and shall sign a written informed consent to confirm their willingness to participate in the trial. The institution where the surgeries are conducted is accredited by “Endocert” (EndoCert certificate, Centres of German Endoprosthetic, German Society for Orthopedics and Traumatology), which supervises and certifies the quality of the surgical procedures. The enrolment in the study will not impact or change the standards used in the management of the patients at our institution.

### Randomisation and blinding

The present study is a protocol for a single-blind randomised controlled trial in which each group of participants is exposed to only one of the study interventions. Patients will be randomly allocated to robotic-assisted TKA or to conventional freehand TKA. All patients in whom a TKA is indicated will be sequentially allocated in a 1:1 ratio to surgeons who perform robotic-assisted TKA or to those who perform conventional freehand TKA at the time of their outpatient appointment. All patients, irrespective of their allocation, will follow the same clinical, imaging, and anaesthesiologic pre- and post-surgical pathways. Patients will be blinded to the allocation until the first postoperative days. Surgeons and personnel involved in the clinical management of the patients will be unblinded to the allocation. Data curacy and collection will be conducted by two assessors blinded to group allocation and not involved in the clinical management of the patients. Assessors will retrieve patient data at the following follow-up times: admission, perioperatively (from access to discharge), at 6 weeks, 12 months, and every 24 months postoperatively. Data from patients will be collected once the learning curve has been reached.

### Eligibility criteria

The inclusion criteria are: (1) age above 18, (2) ability to consent, (3) symptomatic knee osteoarthritis stage II to IV according to the Kellgren-Lawrence classification [16] (Table 1). The exclusion criteria are: (1) acute or chronic inflammatory diseases, (2) neoplastic diseases, (3) pregnancy and lactation, (4) uncontrolled coagulopathy, (5) abnormal cell count, (6) severe peripheral neuropathy, (7) vascular diseases, (8) peripheral ulcers, (9) other condition that could influence the results of the present study.

**Table 1:** Kellgren Lawarence classification

Stadium	Grade of OA	Description
0	No evidence of OA	No radiologic evidence of OA
I	Minimal OA	Doubtful narrowing of joint space and possible osteophytic lipping
II	Mild OA	Definite osteophytes and possible narrowing of joint space
III	Moderate OA	Moderate multiple osteophytes, definite narrowing of joint space, some sclerosis, and possible deformity of bone ends
IV	Severe OA	Large osteophytes, marked narrowing of joint space, severe sclerosis, and definite deformity of bone ends

### Outcomes of interests

**Fig. 1:**
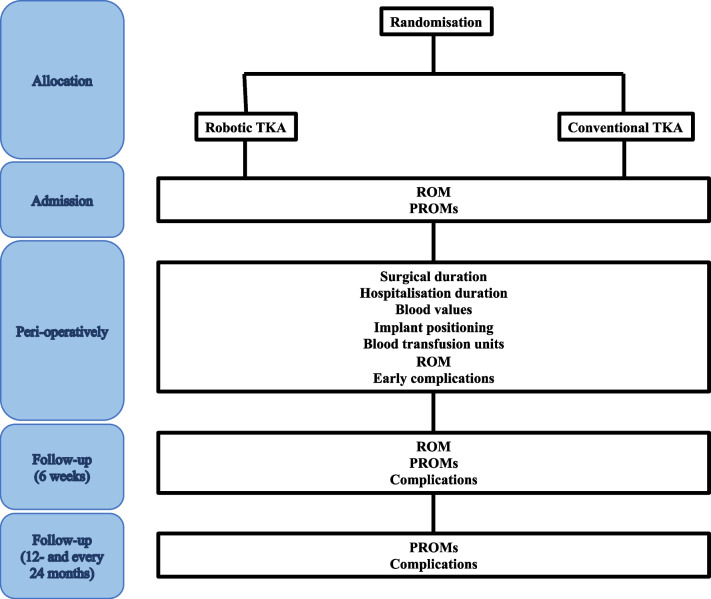
Set up of follow-ups and data extraction

The primary outcome of interest is to compare robotic-assisted TKA using CORI robotic system versus conventional freehand arthroplasty. The surgical duration, length of the hospitalisation, blood analyses, implant positioning, blood units transfused, ROM, PROMs, and complications will be collected from both groups and compared as shown in Fig. 1.

**Fig. 2:**
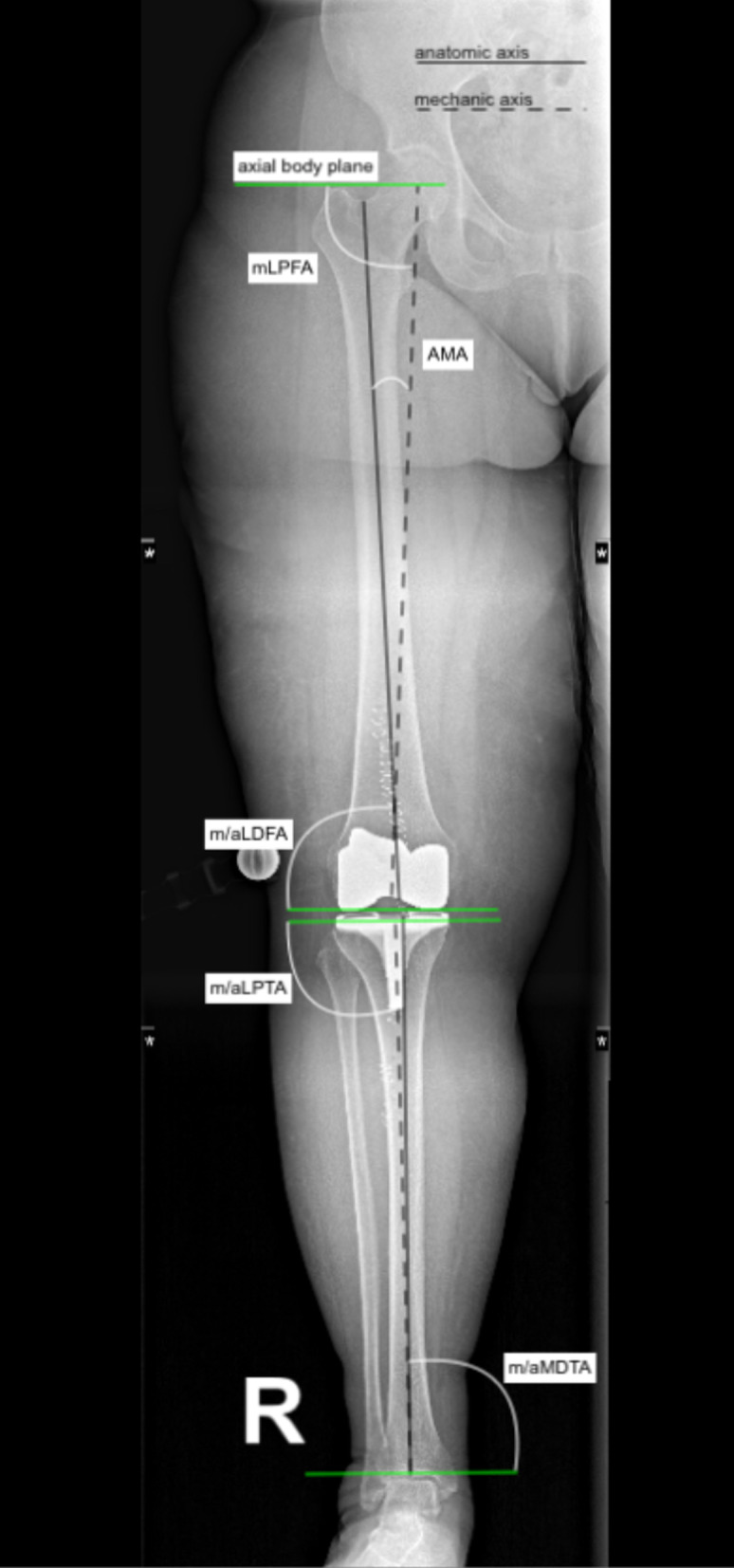
Imaging references, illustrating the main axes and reference angles

The second outcome of interest will be to compare implant positioning between robotic and conventional TKA. Implant positioning will be evaluated using anteroposterior plain radiographs of the leg using the software MediCAD Knie 2D (mediCAD Hectec GmbH, Altdorf, Germany), which is used to perform surgical planning of hip, knee, shoulder, trauma and spine surgery. The imaging references to assess implant positioning are shown in Fig. 2. An explanation of the main imaging references used is shown in Table 2.

**Table 2:** Imaging parameters

Abbreviation	Definition	Mean	Range	Description
mLDFA	Mechanical lateral distal femoral angle	88°	85–90	Angle between the lateral femur and the proximal femoral joint
mLPFA	Mechanical lateral proximal femoral angle	90°	85–95	Angle between the mechanical femoral shaft axis and the orientation line through the centre of the femoral head and the tip of the greater trochanter
m/aLDTA	Mechanical/anatomical lateral distal tibial angle	89°	86–92	Angle between the mechanical axis of the tibia and the tibial plafond, measured on the lateral side
m/aMPTA	Mechanical/anatomical medial proximal tibial angle	87°	85–90	Angle between the mechanical axis of the tibia and the tibial plateau knee joint line, measured on the medial side
JLCA	Joint line convergence angle	1.5° medial	0–3	Interaction of the intra-articular deformity arising from the osteoarthritis and the surrounding soft tissue laxity
MAD	Mechanical axis deviation	10 mm medial	3–17	Distance from the centre of the knee joint and the line of the mechanical axis
AMA	Anatomical-mechanical-angle	6°	5–7	Angle between anatomical and mechanical femoral axes

### Data to be collected

**Fig. 3:**
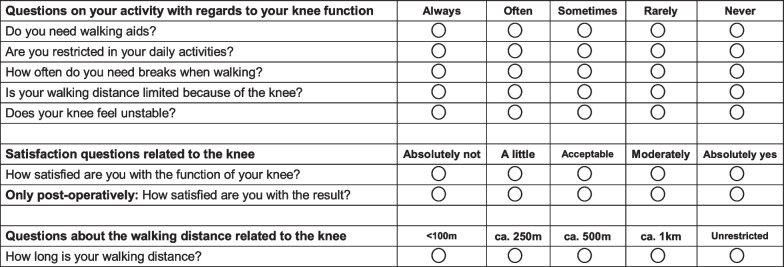
Likert-like questionnaire

On admission, the demographic information (age at surgery, BMI, sex), stage of OA according to the Kellgren-Lawrence classification [16], PROMs, and blood values of each patient will be collected on admission. Concerning the blood values, the haematocrit, haemoglobin, and C-reactive protein (CRP) will be collected preoperatively, on the first and fifth postoperative days. The blood tests will be conducted on admission, postoperative day (POD) 1 and 5 by dedicated healthcare personnel. To assess ROM, the neutral-zero method shall be used [17], using a standard baseline plastic 360-degree plastic pocket goniometer with flexion-hyper extension gauge (ProHealthcareProducts.com, Park City, US). ROM surgery will be assessed by moving the foot from a neutral starting position (neutral position in extension) in flexion and extension. ROM data will be collected on admission, on POD 1, 2, 3, 4, 5, and at each follow-up appointment by medical personnel who has not performed the index surgery. The German version of the Western Ontario and McMaster Universities Osteoarthritis Index (WOMAC) [18] and the visual analogue rating scale of health-related quality of life [19] will be used to rate the clinical outcomes. The patients will complete the questionnaires at admission and at each post-operative follow-up appointment. To evaluate the activity level of the patients included in the trial, the German version of the Tegner score will be administered [20]. In addition, a further seven questions Likert-like questionnaire shall be administered to all patients. This questionnaire is routinely used at our institution to rate the function of the knee in patients who undergo TKA. The questionnaire enquires about the activities related to the knee, post-operative overall patient satisfaction, patient satisfaction with the function of the knee, and information concerning walking distance. The Likert-like questionnaire is reported in Fig. 3, and the German version is available in Additional file 1.

Concerning complications, the number of wound healing complications, haematomas, anterior knee pain, periprosthetic fractures, deep infections, aseptic loosening, deep vein thromboses, and surgical revision will be collected at discharge and each follow-up.

### Surgical procedures and rehabilitation protocol

All patients shall receive a 1.5 g single administration of intravenous cefuroxime at induction of anaesthesia. A femoral nerve block shall be used for pain control and maintained for 48 h. A tourniquet 120 mmHg over the systolic arterial pressure shall be used. All surgeries will be performed using a standard medial parapatellar approach. A restricted kynematic alignment will be performed to all TKA. All components will be implanted following manufacturer instructions using the Smith & Nephew Legion Genesis II, with a posterior stabilised polyethylene liner insert. Both femoral and tibial implants will be cemented using Palacos cement (Heraeus Medical GmbH, Wehrheim, Germany). At the end of the procedure, 1 g of tranexamic acid will be injected intra-articularly. One closed suction deep drain and one open suction subcutaneous drain shall be used for the first 48 h. Anti-thrombotic prophylaxis with Rivaroxaban, 10 mg daily for 6 weeks, will start 12 h after the index procedure. Physiotherapy will follow standard protocols [21]. Patients will be followed by a team of physiotherapists during hospitalization from the first postoperative day. In the absence of complications or other medical reasons who prevent discharge, the standard length of hospitalisation at our institution is 5 days. Moreover, from POD-2, each patient will undergo two sessions of physiotherapy daily using continuous passive motion (CPM) for 60 min per session to flex and extend the knee joint. The physiotherapist will increase the range of motion at each. Patients shall be discharged when they shall have reach at least 80° of flexion. Starting from POD-2, patients will start to walk under physiotherapeutic supervision, and on POD-4 they shall start to ascend and descend stairs. A personalised outpatient or inpatient rehabilitation program will be set for every patient for a minimum of 3 weeks. Deviation from the planned surgical procedure and rehabilitation protocol shall warrant exclusion from the study.

### Sample size evaluation

Assuming a true difference in means between the test and the reference group of 5 units, and a pooled standard deviation of 20 units, the study would require a sample size of 198 patients for each group (i.e. a total sample size of 396, assuming equal group sizes), to achieve a power of 80% and a level of significance of 5%, for declaring that the robotic TKA is superior to traditional TKA at 10 units margin of superiority, assuming a minimum clinically important difference for the WOMAC total subscale after TKA of 10 points [22].

### Statistical analysis

All statistical analyses will be conducted by the main author (FM). For descriptive statistics, arithmetic mean and standard deviation shall be used. The unpaired t-test will be performed to assess baseline comparability, with values of *P* > 0.05 considered satisfactory. Data items will be collected by the assessors prospectively and entered into Microsoft Excel Spreadsheet Software version 2020 (Microsoft Corporation, Redmond, US). To compare robotic TKA versus freehand TKA, the IBM SPSS version 25 will be used. For continuous and binary data, the mean difference (MD) and odds ratio (OR) effect measures will be used. Standard error and 95% of confidence interval will be also evaluated. The null hypothesis will be that no difference between the two techniques exists. Both *χ*^2^ and unpaired *t*-test will be performed to evaluate whether the null hypothesis can be refused. Values of *P* < 0.05 were considered statistically significant. To evaluate the accuracy of implant positioning of robotic versus conventional TKA with regards to the pre-operative planning the standard deviation from the optimal implant alignment according to the patient anatomy will be evaluated using the IBM SPSS version 25.

## Discussion

Changes in surgical technique in experienced surgeons are always a challenge. The advantages of robotic surgery over conventional freehand TKA are still unclear, and evidence is missing. Therefore, the present randomised controlled trial wishes to evaluate the effect of the introduction of robotics in the arthroplasty of the knee in a routine clinical setting. According to the manufacturer, CORI is an intelligent platform which supports robotics, software, intelligent tools and data, and surgeon-controlled imageless intelligence with fast mapping. CORI delivers real-time planning and joint space assessment to improve soft tissue balance. CORI is believed to promote high accuracy of bone resection and implant alignment, reducing variation in component position [23–25]. However, these features have not yet been validated in clinical practice, and evidence is lacking.

Data from patients will be collected once the learning curve has been reached. The learning curve represents the relationship between how proficient surgeons will be in the performance of robotic TKA and the amount of experience they have. Data on the surgical duration will be collected. Surgical duration is considered as the minutes elapsed from incision to complete suture of the wound. The learning curve is considered the number of robotic TKA necessary to reach a plateau in the curve of the surgical duration. The plateau in surgical time implies that the surgeons’ skills do not improve substantially with each further operation, with less new expertise gained. Robotic TKA using CORI is believed to reduce surgical time. The CORI software offers a surgical workflow which improves efficiency and ease of use, further shortening the learning curve, with a 72% reduction in required data point collection with automatic landmark capture, 40% fewer work steps, faster surface model generation, and a 29% faster bony resection [26]. The evidence on the learning curve of robotic TKA is limited, and the literature would benefit from further investigations. However, the learning curve is dependent on assumptions made about performance, and many variables impact learning and future performance. The predictive value of the learning curve to predict the overall performance of larger groups should be considered with caution, as the assumptions are made on heterogeneous variables, including surgeon motivation, workplace dynamics, and training resources. Further investigations should clarify the learning curve of robotic TKA using CORI and whether previous knowledge or experience impacts the learning curve.

Accurate pre-operative planning to determine the correct implant size and position is an important aspect which supports the surgeon pre- and peri-operatively. It is regarded as a vital step to successful component implantation, which may increase implant survival and reduce complications associated with surgery [27, 28]. Furthermore, pre-surgical templating is also important from an economic point of view, as a correct estimation of component sizes can avoid the waste of expensive components. However, it is unclear whether robotic TKA will produce greater accuracy in preoperative planning, and clinical investigations are missing.

Approximately 2000 TKAs are performed each year at our institution, which has been accredited by “Endocert” since 2016. The EndoCert initiative represents the first worldwide certification system of medical centres for total joint replacement and was established in Germany in 2012. The EndoCert aims to maintain quality standards in primary and revision arthroplasty. The associated centres also develop and define standards as well as treatment processes, and they are subject to continuous re-certification [29, 30]. All surgeries shall be conducted by six surgeons in a highly standardized fashion. All surgeons have obtained the certificate of the senior operator of EndoCert, and are well beyond their learning curve, having each performed more than 250 knee arthroplasties.

Some limitations of the present protocol must be acknowledged. The use of the CORI system during TKA requires special instrumentation, training and organisation. Surgery with CORI might initially require a longer time, and the surgical slots will be reserved for a longer time. Therefore, the healthcare staff involved in the clinical management of the patients will not be blinded, increasing the risk of performance bias. Patients will be informed in regard to the nature of the surgery (freehand or robotic) during their postoperative inpatient stay. According to current German legislation, all patients will receive a discharge letter with a description of the surgical intervention. In this instance, patients will have to be aware of their allocation, impacting on detection bias. The Likert-like questionnaire used in the present study is routinely administered at our institution to patients who undergo TKA to rate the function of the knee. Investigations are ongoing to validate this questionnaire in the clinical setting.

### Supplementary Information

**Additional file 1.** German version of the Likert-like questionnaire.

## Data Availability

No dataset has been generated during the current investigation.

## Abbreviations

TKA: Total knee arthroplasty
CPM: Continuous passive motion
ROM: Range of motion
PROMs: Patient-reported outcome measures
CRP: C-reactive protein
WOMAC: Western Ontario and McMaster Universities Osteoarthritis Index
VAS: Visual analogue scale
MD: Mean difference
OR: Odd ratio
