# Supplementary material for: Robotic-assisted total knee arthroplasty in clinical practice: protocol for a randomised controlled trial
Source: J Orthop Surg Res. 2023 Aug 25;18:623. doi: 10.1186/s13018-023-04101-z (PMC10464371; doi:10.1186/s13018-023-04101-z)
Supplement: Supplementary file 1 — Additional file 1. German version of the Likert-like questionnaire. [file 13018_2023_4101_MOESM1_ESM.pdf]

| **Fragen zu Aktivitäten bezogen auf das Knie** | **Immer** | **Oft** | **Manchmal** | **Selten** | **Nie** |
|---|---|---|---|---|---|
| Brauchen Sie Gehhilfen | ◯ | ◯ | ◯ | ◯ | ◯ |
| Sind Sie in Ihren Alltagsaktivitäten eingeschränkt | ◯ | ◯ | ◯ | ◯ | ◯ |
| Wie oft brauchen Sie Pausen beim Laufen | ◯ | ◯ | ◯ | ◯ | ◯ |
| Ist Ihre Gehstrecke wegen des Knies eingeschränkt | ◯ | ◯ | ◯ | ◯ | ◯ |
| Fühlt sich Ihr Knie instabil an | ◯ | ◯ | ◯ | ◯ | ◯ |
| | | | | | |
| **Fragen zur Zufriedenheit bezogen auf das Knie** | **Gar nicht** | **Minimal** | **Akzeptabel** | **Moderat** | **Sehr** |
| Wie zufrieden sind Sie mit der Funktion Ihres Knies | ◯ | ◯ | ◯ | ◯ | ◯ |
| **Nur nach der OP**: Wie zufrieden sind Sie mit dem Ergebnis | ◯ | ◯ | ◯ | ◯ | ◯ |
| | | | | | |
| **Fragen zur Gehstrecke bezogen auf das Knie** | **Kürzer als 100m** | **ca. 250m** | **ca. 500m** | **ca. 1km** | **Uneingeschränkt** |
| Wie lang ist Ihre Gehstrecke | ◯ | ◯ | ◯ | ◯ | ◯ |
